# Role of CARD9 in Cell- and Organ-Specific Immune Responses in Various Infections

**DOI:** 10.3390/ijms25052598

**Published:** 2024-02-23

**Authors:** Ji Seok Lee, Chaekyun Kim

**Affiliations:** 1 Laboratory of Leukocyte Signaling Research, Department of Pharmacology, Inha University School of Medicine, Incheon 22212, Republic of Korea; jiseok@inha.edu; 2 BK21, Program in Biomedical Science & Engineering, Inha University, Incheon 22212, Republic of Korea

**Keywords:** caspase recruitment domain-containing protein 9 (CARD9), microbial infections, cytokines, chemokines, infiltration, neutrophils

## Abstract

The caspase recruitment domain-containing protein 9 (CARD9) is an intracellular adaptor protein that is abundantly expressed in cells of the myeloid lineage, such as neutrophils, macrophages, and dendritic cells. CARD9 plays a critical role in host immunity against infections caused by fungi, bacteria, and viruses. A CARD9 deficiency impairs the production of inflammatory cytokines and chemokines as well as migration and infiltration, thereby increasing susceptibility to infections. However, CARD9 signaling varies depending on the pathogen causing the infection. Furthermore, different studies have reported altered CARD9-mediated signaling even with the same pathogen. Therefore, this review focuses on and elucidates the current literature on varied CARD9 signaling in response to various infectious stimuli in humans and experimental mice models.

## 1. Introduction

The activation of the immune system against invading pathogens such as fungi, bacteria, and viruses is a critical process in host defense. These pathogens are recognized by pattern recognition receptors (PRRs) expressed on immune cells, which in turn activate the immune response. There are four main types of PRRs: Toll-like receptors (TLRs), c-type lectin receptors (CLRs), retinoic acid-inducible gene I (RIG-I)-like receptors (RLRs), and nucleotide oligomerization domain (NOD)-like receptors (NLRs) [1]. Other PRRs include AIM2-like receptors (ALRs), peptidoglycan-binding proteins (PGBPs), the signaling lymphocytic activation molecule family (SLAMF), and oligoadenylate synthetase (OAS)-like receptors (OLRs) [2]. TLRs and CLRs are transmembrane proteins responsible for extracellular signaling, while RLRs and NLRs are cytoplasmic proteins responsible for intracellular signaling. Some TLRs (TLR1, 2, 4, 5, 6, and 10) are expressed on the surface of immune cells as hetero or homodimers and primarily recognize the membrane components of microorganisms, such as lipids, lipoproteins, and proteins. Others (TLR3, 7, 8, and 9) are expressed primarily as homodimers that recognize the nucleic acids of microorganisms [3]. CLRs (dectin-1, dectin-2, mincle, mannose receptors, and dendritic cell-specific intracellular adhesion molecule (ICAM)-3-grabbing non-integrins (DC-SIGN)) respond to pathogens primarily through the recognition of carbohydrate structures such as mannose, fucose, and glucan [4]. NLRs (NOD1 and 2) are major sensors of bacterial peptidoglycans and are important for tissue homeostasis and host defense against bacterial pathogens [5]. RLRs are the key PRRs that are essential for recognizing RNA virus infections [6]. PRRs trigger the signaling pathways such as the mitogen-activated protein kinases (MAPKs), nuclear factor kappa-light-chain-enhancer of activated B cells (NF-κB), interferon regulatory factors, and inflammasomes, and subsequently the downstream immune effector modules including cytokines, antimicrobial peptides, and antibodies [2].

The caspase recruitment domain-containing protein 9 (CARD9) is an adaptor protein that is abundantly expressed in myeloid cells, including neutrophils, macrophages, and dendritic cells (DCs). CARD9 comprises 536 amino acids with an estimated molecular weight of 62.3 kDa, and it is mapped to the chromosomal region 9q34.3 [7,8]. CARD9 contains a CARD domain at the N-terminus that mediates homology interactions between CARD9-containing molecules and a coiled-coil region at the C-terminus that functions as an oligomerization domain [8,9]. The levels of CARD9 expression vary in different organs; it is abundant in organs rich in cells associated with immune response such as the bone marrow, spleen, lung, and lymph node but it is not expressed in organs such as kidney, liver, intestine, colon, and brain [8,10].

CARD9 is required for the downstream signal transduction of PRRs, including TLRs and CLRs. CLRs such as dectin-1, dectin-2, and mincle utilize the signaling pathway involving spleen tyrosine kinase (Syk)/CARD9 [11,12]. Upon pathogen recognition via the CLRs, Syk is phosphorylated and induces the activation of protein kinase Cδ, which mediates the recruitment and activation of CARD9. Activated CARD9 binds to B-cell lymphoma/leukemia (BCL) 10 and mucosa-associated lymphoid tissue lymphoma translocation protein (MALT) 1 to form the CARD9-BCL10-MALT1 (CBM) complex, which activates NF-κB and MAPKs. These pathways lead to the transcription of genes involved in inflammation and pro-inflammatory cytokine and chemokine production. CARD9 is also involved in cell invasion and induction of oxidative stress [13].

Cytokines are small proteins that are released by immune cells as well as a variety of other cells (fibroblasts, endothelial cells, epithelial cells, etc.) in the body in response to inducing stimuli [14]. They can be grouped by structure into families: Interleukin (IL)-1 family, interferon (IFN) family, tumor necrosis factor (TNF), and chemokines [15,16]. The immune cells secrete cytokines in response to activation by PRRs. The cytokines (IL-1β, IL-6, IL-17, IFN-γ, and TNF-α) and chemokines (CXCL1 (keratinocyte chemoattractant, KC), CXCL2 (macrophage inflammatory protein, MIP-2), and CXCL5) are the primary cytokines regulated by CARD9 [17]. IL-1β and IL-6 are produced primarily by macrophages and activate lymphocytes and inflammatory responses. IL-17 is secreted by the T cells and stimulates lymphocytes to induce inflammatory responses and activate cytokine and chemokine production. IFN-γ is produced primarily by T cells and natural killer (NK) cells and stimulates the neutrophil, monocyte, and macrophage functions. TNF-α is produced primarily by macrophages and activates phagocytes. Chemokines control innate immune cell trafficking. CXCL1, CXCL2, and CXCL5 induce neutrophil migration [18].

The CARD9-mediated responses to pathogenic stimuli exhibit variability contingent upon the type of stimulus, the cellular context, and the affected organ. This review aims to elucidate the role of CARD9 in infections caused by various pathogens.

## 2. Effects of CARD9 on the Immune Response to Fungal Infections

CARD9 is considered a crucial activator of the immune response against fungi [17]. The most common infectious fungi include *Aspergillus*, *Candida*, *Cryptococcus*, *Exophiala*, *Mucor*, and *Pneumocystis* (Table 1). Recognition of fungi primarily relies on CLRs, which utilize the signaling pathway involving Syk and the CBM complex, subsequently leading to activation of the NF-κB and MAPK pathways [11,12].

### 2.1. Aspergillus fumigatus

*Aspergillus fumigatus* (*A. fumigatus*) is a filamentous mold of the genus *Aspergillus*. It is one of the most common and clinically important species in the genus. It is generally harmless to healthy individuals but can pose a serious health risk to individuals with weakened immune systems or underlying respiratory conditions [19].

In CARD9-deficient patients with an *A. fumigatus* infection, there was a decrease in the production of cytokines (TNF-α, granulocyte-macrophage colony-stimulating factor (GM-CSF), IFN-γ, IL-1β, IL-6, IL-17A, and IL-22) in the peripheral blood mononuclear cells (PBMCs) and T helper (Th) cells including the Th1, Th17, and Th22 cells [20,21]. However, in these patients, the phagocytosis and killing capacity of neutrophils were not impaired, though they exhibited impaired extrapulmonary infiltration [20]. These results are consistent with results from studies on CARD9-deficient mice, wherein a decrease in cytokine (IL-1β, IL-6, IL-22, TNF-α, and IFN-γ) and chemokine (CXCL1, CXCL2, and CXCL5) levels was observed except for IL-17A levels, which were found to be normal [21,22,23]. Further, the CARD9-deficient mice were also observed to have a reduced infiltration of neutrophils and T cells. Moreover, the fungal burden in the footpads and lungs was found to be higher in CARD9-deficient mice compared to the wild type [21].

In summary, the immune response to *A. fumigatus* is impaired, with a decrease in cytokine and chemokine production as well as an infiltration of inflammatory cells in CARD9-deficient animal models and patients. It is known that *A. fumigatus* is recognized by dectin-2, and Syk plays an essential role in NF-κB activation and reactive oxygen species (ROS) production mediated by dectin-2 activation following *A. fumigatus* stimulation [24]. Therefore, CARD9 may play a role via the dectin-2/Syk signaling pathway in *A. fumigatus* infection.

### 2.2. Candida albicans

*Candida albicans* (*C. albicans*) is an opportunistic pathogenic fungus commonly found as part of the normal microbiome in the human digestive tract and genital organs, and could cause systemic candidiasis under certain specific host conditions. The cell wall of *C. albicans* is composed of β-glucan, mannans, and cell wall proteins, which are recognized by CLRs [25]. While a deficiency of CLRs does not impair survival and neutrophil accumulation [26,27], the deficiency of dectin-1 and dectin-2 during *C. albicans* infection results in increased fungal burden in the brain [27].

Impaired immune responses to *C. albicans* have been reported in patients with CARD9 deficiency and CARD9-deficient mice. During a *C. albicans* infection in CARD9-deficient patients, the production of cytokines (IL-1β, IL-6, IL-17, IL-22, TNF-α, GM-CSF, and IFN-γ) and chemokines (CXCL1, CXCL2, and CXCL8) were reduced in PBMCs [20,28,29]. The production of IL-8 was also decreased in *C. albicans*-stimulated neutrophils of CARD9-deficient patients [29]. Moreover, the proportions of Th17 and Th22 cells were decreased, but the proportion of Th1 cells was not affected [28]. Consistent with findings in patients, bone marrow-derived macrophages (BMDMs) from CARD9-deficient mice stimulated with *C. albicans* showed a decrease in the production of cytokines (CCL3 (MIP-1α), CXCL1, CXCL2, and TNF-α [30].

In both CARD9-deficient patients and mice, phagocytosis of *C. albicans*, CLR (dectin-1 and dectin-2) expression, and ROS production remained intact [28,29,30]. However, the killing capacity against *C. albicans* was impaired [29,31,32]. Moreover, the accumulation of neutrophils in the infected central nervous system (CNS) was significantly reduced in CARD9-deficient patients [31], and this reduction was associated with a decrease in several chemokines (CXCL1, CXCL2, and CXCL5). In *C. albicans*-infected CARD9-deficient mice, neutrophil accumulation was reduced in the brain but increased in the kidneys [27,31]. However, the fungal burden was increased in the spleen, kidneys, liver, and brain of CARD9-deficient mice [27,30,31]. The kidneys are recognized as a primary target organ for candidiasis and renal failure is responsible for 30–50% of deaths in humans with candidiasis [33]. Collectively, these results suggest that CARD9 plays a species- and organ-specific role in the accumulation of neutrophils, and CARD9 can play a CLR-independent role in the host defense against *C. albicans* infection.

In the kidneys of CARD9-deficient mice, IL-1α, IL-1β, IL-6, CCL2 (monocyte chemoattractant protein, MCP-1), and TNF-α were increased, while the levels of IFN-γ were similar to the wild type [30]. The increase in cytokines may be the result of an excessive inflammatory response. The presence of myeloperoxidase (MPO), an enzyme found primarily in neutrophils that plays a crucial role in the defense system against microorganisms, was increased in the kidneys of CARD9-deficient mice [30], suggesting an increased infiltration of neutrophils. Furthermore, an increased fungal burden in the kidneys, liver, and lungs of CARD9-deficient mice infected with *C. albicans* and an increase in mortality has been reported [34].

Taken together, these results suggest that during a *C. albicans* infection, CARD9 is required for cytokine and chemokine production in PBMCs and neutrophils as well as for the killing capacity of neutrophils. However, CARD9 is not associated with phagocytosis, ROS production, or expression of CLRs in PBMCs and neutrophils. Moreover, the excessive inflammatory response in the kidneys of CARD9-deficient mice is likely a result of signals unrelated to CARD9.

### 2.3. Candida parapsilosis

Like other *Candida* species, *Candida parapsilosis* (*C. parapsilosis*) exists in the human body as a commensal organism; and in most cases, causes no harm. However, *C. parapsilosis* can become an opportunistic pathogen and cause infections, particularly in individuals with compromised immune systems or those with medical implants [35].

During *C. parapsilosis* infection in CARD9-deficient mice, the production of TNF-α and chemokines (CXCL1, CXCL2, and CCL3) was reduced and the activation of NF-κB was impaired in BMDMs [30]. The phagocytic and killing capacities of BMDMs of CARD9-deficient mice were not impaired, but the fungal burden increased in the spleen, kidney, liver, and brain [30]. Although it is unclear which receptors are involved in *C. parapsilosis* recognition, *C. parapsilosis* induces NF-κB activation through the Syk/CARD9 pathway and regulates cytokine production in BMDMs [30]. Interestingly, unlike in *C. albicans*, there was no increase in cytokines (IL-1β, IL-6, CCL2, and TNF-α) and MPO in the kidneys of CARD9-deficient mice infected with *C. parapsilosis*. However, an increase in IFN-γ was observed, which is contrary to what was observed during a *C. albicans* infection [30]. This suggests that fungi within the same genus elicit different immune responses, and the role of CARD9 varies depend on the stimulus.

### 2.4. Candida tropicalis

*Candida tropicalis* (*C. tropicalis*) is a yeast species belonging to the non-albicans *Candida* group but is closely related to *C. albicans*. It is generally regarded as an opportunistic pathogen that commonly causes infections in neutropenic hosts and can be transmitted to the peripheral organs through the bloodstream [36].

CARD9-deficient mice infected with *C. tropicalis* revealed reduced survival and an increased fungal burden in the kidney, brain, and liver, but not in the spleen [37]. The killing ability of *C. tropicalis* did not vary in the monocytes from wild-type and CARD9-deficient mice. However, it was reduced in CARD9-deficient BMDMs [37,38]. The expression of IL-1β, IL-6, and IL-17 did not differ in the kidneys of CARD9-deficient and wild-type mice. However, the production of TNF-α was reduced, suggesting that the reduced host defense in CARD9-deficient mice did not involve Th17 cells or IL-17, and was associated with reduced TNF-α [37]. Mice lacking dectin-1 are more susceptible to *C. tropicalis* infection [37]. Therefore, it is likely that dectin-1 acts as the primary receptor for *C. tropicalis* recognition, and CARD9 is essential for signal transduction and downstream cellular response of dectin-1, leading to TNF-α production.

In summary, the deficiency of CARD9 in *C. tropicalis* infections leads to uncontrolled fungal growth and tissue-specific damage due to a defect in TNF-α production, rather than the involvement of IL-17 and Th17 cells. The reduced immune response is not associated with the accumulation of neutrophils and monocytes [37]. Moreover, TNF-α produced in a CARD9-dependent manner can enhance the killing activity of neutrophils but does not affect monocytes [37].

### 2.5. Cryptococcus neoformans

*Cryptococcus neoformans* (*C. neoformans*) causes infections in individuals with weakened immune systems. It caused life-threatening infections of the CNS in immunocompromised patients [39]. CARD9 deficiency resulted in impaired immune responses to *C. neoformans* infection [40,41,42]. The fungal burden was increased in the lungs and spleen, but not in the brain, and survival was reduced in CARD9-deficient mice [40,42]. However, the leukocyte infiltration was not impacted by CARD9 deficiency [40]. In contrast, the accumulation of neutrophils was significantly increased in CARD9-deficient mice [42]. The production of Th2-type cytokines (IL-4, IL-5, IL-6, IL-13, and IL-10) was increased, while that of IL-17 was decreased in the lungs of CARD9-deficient mice [40]. Moreover, the chemokine production was increased in the same study. However, in another study, the expression of chemokines (CCL4, CCL5, CXCL9, and CXCL10) that attract NK cells and memory T cells was reduced, and the cytokines critical for Th17 cell differentiation (IL-23p19, transforming growth factor (TGF)-β) and retinoic acid receptor-related orphan receptor (ROR-γt) were decreased in CARD9-deficient mice [42].

Anticryptococcal activity was impaired in CARD9-deficient macrophages, but not in CARD9-deficient DCs [40]. Moreover, CARD9 was required for M1 macrophage activation. CARD9 also contributes to the phagocytosis of *C. neoformans* by bone marrow-derived DCs (BMDCs), which is mediated by dectin-2 and CARD9 and occurs via actin polymerization [41]. However, dectin-2-deficient mice showed normal survival when they were infected with *C. neoformans*, while they showed increased mortality to *C. albicans* infection [41,43]. It appears that dectin-1 is not required for the recognition and signaling of *C. neoformans* [44]. In addition to dectin-2, the mannose receptors and DC-SIGN have also been reported to recognize *C. neoformans* [45,46].

### 2.6. Exophiala spinifera

*Exophiala spinifera* (*E. spinifera*) is a species of black yeast-like fungus belonging to the genus *Exophiala*. These fungi are known for their ability to thrive in extreme environments and can be found in various habitats, including soil, water, and decaying organic matter. *Exophiala* species are known to be opportunistic human pathogens, capable of causing infections in individuals with compromised immune systems. *E. spinifera* has been associated with subcutaneous and systemic infections [47].

During *E. spinifera* infection, PBMCs from CARD9-deficient patients expressed lower levels of cytokines (IL-1β, IL-6, IL-17, IL-22, and TNF-α) and chemokines (CXCL1, CXCL2, and CXCL8), and decreased activation of NF-κB than healthy PBMCs [28]. However, phagocytosis and ROS production were not affected by CARD9 deficiency [28]. Patient samples showed a significant reduction in Th17 and Th22 cells, while the relative proportion of Th1 cells was comparable to that observed in healthy controls [28]. CARD9-deficient humans have reduced cytokine secretion in response to dectin-1 and NOD2 ligands [28]. Since CARD9 is involved in the dectin-1 and NOD2 signaling pathways, the impaired immune response to *E. spinifera* may be due to impairment of these pathways. In addition, cytokines (IL-1β, IL-6, IL-17A, IL-22, IFN-γ, and TNF-α) and chemokines (CXCL1 and CXCL2) were decreased in the footpads of CARD9-deficient mice [28]. As the levels of cytokines and chemokines decreased, there was a reduction in neutrophil infiltration and an increase in the fungal burden in the footpads of *E. spinifera*-infected mice [28]. Moreover, CARD9-deficient BMDMs stimulated with *E. spinifera* show diminished activation of the NF-κB and p38 MAPK pathway [28].

### 2.7. Mucor irregularis

*Mucor irregularis* (*M. irregularis*) belongs to the *Mucoraceae* family. It is commonly known as the “black bread mold” or simply “mucor mold.” Like other molds in the *Mucor* genus, *M. irregularis* is a fast-growing, saprophytic fungus that can be found in various environments, especially on decaying organic matter [48].

CARD9-deficient mice exhibited increased susceptibility to *M. irregularis* infection and impaired cytokine and chemokine production compared to the wild type [49]. Cytokines (IL-1β, IL-6, IL-12p70, IL-17A, IL-23, IFN-γ, and TNF-α) and chemokines (CXCL1 and CXCL2) were reduced in CARD9-deficient mice in response to an *M. irregularis* infection, but IL-4, IL-10, IL-17A, and IFN-γ increased over time to the levels of the wild type [49]. Neutrophils of CARD9-deficient mice showed reduced formation of neutrophil extracellular traps (NETs), and BMDMs showed impaired NF-κB activation [49]. Furthermore, BMDCs showed reduced Th1/Th17 cell differentiation, resulting in the decreased production of the cytokines IL-1β, IL-12p70, and IL-23 [49].

In summary, in response to *M. irregularis* infection, CARD9 regulates fungal clearance as well as the production of cytokines and chemokines. It is also required for the proper functioning of immune cells, including neutrophils, macrophages, and DCs.

### 2.8. Pneumocystis jirovecii (P. murina and P. carinii)

Pneumocystis pneumonia is a fungal infection caused by *Pneumocystis jirovecii*, which causes infection in humans with weakened immune responses. The species *P. carinii* and *P. murina* are members of the genus associated with rats and mice [50].

During *P. murina* infections, the production of cytokines (IL-1β, IL-6, and TNF-α), MPO, and granulocyte colony-stimulating factor (G-CSF) were reduced in the lungs of CARD9-deficient mice [51]. The infiltration of alveolar monocytes/macrophages, neutrophils, and DCs was also decreased. However, there were no significant differences in the survival rates in CARD9-deficient mice [51]. Moreover, the Th cell-derived cytokines (IL-4, IL-17, and IFN-γ) were not affected by the CARD9 deficiency, which demonstrates that CARD9 is not required for Th cell responses during a pneumonia infection [51]. In BMDMs from the CARD9-deficient mice, the production of IL-6 and IL-12, and the activation of MAPKs were reduced [51]. Furthermore, CARD9-deficient BMDMs exhibited a defect in the markers of M1 (inducible nitric oxide synthase, iNOS) and M2 (Arg-1) macrophages, as well as in the expression of CLRs [51].

These results suggest that CARD9 regulates the production of inflammatory cytokines and the infiltration of immune cells during *Pneumocystis* infections. However, cytokines derived from Th cells, such as IL-4, IL-17, and IFN-γ, are not affected in CARD9-deficient mice. These results represent a different response from those observed in other fungal infections, such as *A. fumigatus* and *C. albicans*.

**Table 1:** Role of CARD9 in fungal infections.

Pathogens: Fungi	Host	Effects of CARD9			Refs.
		Positive	Negative	No Effect	
*A. fumigatus*	H * (c.883C>T) (c.3G>C) (c.819_820insG)	Production of cytokines (IL-1β, IL-6, IL-17A, IL-22, TNF-α, GM-CSF, IFN-γ) Th cell response (Th1, Th17, Th22) Infiltration of PMNs		Phagocytosis of PMNs Killing ability of PMNs Chemotactic capacity of PMNs	[20,21]
	M * (C57BL/6 vs. CARD9^−/−^)	Production of cytokines (IL-1β, IL-6, IL-22, TNF-α, IFN-γ) Production of chemokines (CXCL1, CXCL2, CXCL5) Infiltration of PMNs and T cells Clearance of fungus in footpad and lung		Production of cytokine (IL-17A) Survival Chemotactic capacity of PMNs	[21,22,23]
*C. albicans*	H (c.883C>T) (c.3G>C) (c.170G>A)	Production of cytokines (IL-1β, IL-6, IL-8, IL-17, IL-22, TNF-α, GM-CSF, IFN-γ) Production of chemokines (CXCL1, CXCL2, CXCL5, CXCL8) Th cells response (Th17, Th22) Killing ability of PMNs		Phagocytosis of PMNs and PBMCs ROS production of PMNs Expression of CLRs (dectin-1, dectin-2) Th1 cell response Apoptosis of PMNs [31]	[20,28,29,30,31,32]
	M (C57BL/6 vs. CARD9^−/−^)	Production of cytokine (TNF-α) Production of chemokines (CXCL1, CXCL2, CXCL5, CCL3) Killing ability of PMNs Infiltration of PMNs in the brain [31] Clearance of fungus in the spleen, kidney, liver, and brain Survival Activation of NF-κB	Production of cytokines (IL-1α, IL-1β, IL-6, TNF-α, CCL2) in the kidney Mitochondrial ROS production of PMNs Oxidative phosphorylation activity of PMNs Infiltration of PMNs in the kidney [30,31] Apoptosis of PMNs [32] MPO stain in the kidney	Phagocytosis of BMDMs and PMNs Killing ability of BMDMs ROS production of PMNs	[27,30,31,32,34]
*C. parapsilosis*	M (C57BL/6 vs. CARD9^−/−^)	Production of cytokine (TNF-α) Production of chemokines (CXCL1, CXCL2, CCL3) Activation of NF-κB Clearance of fungus in the kidney, spleen, liver, and brain	Production of cytokines (IL-1α, IFN-γ) Infiltration of immune cells	Production of cytokines (IL-1β, IL-6, TNF-α) in the kidney Production of chemokine (CCL2) in the kidney MPO production in the kidney Phagocytosis of BMDMs Killing ability of BMDMs	[30]
*C. tropicalis*	M (C57BL/6 vs. CARD9^−/−^)	Production of cytokine (TNF-α) Clearance of fungus in the kidney, brain, and liver Killing ability of macrophages Survival M2 differentiation	Infiltration of immune cells	Production of cytokines (IL-6, IL-1β, IL-23, IL-17A) Production of chemokines (CXCL1, CXCL2, CXCL5) Clearance of fungus in the spleen Killing ability of PMNs Infiltration of PMNs and monocytes	[37,38]
*C. neoformans*	M (C57BL/6 vs. CARD9^−/−^)	Production of cytokines (IL-17, IL-12p70, IL-23p19, TGF-β, ROR-γt) Production of chemokines (CCL3, CCL4, CCL5, CXCL9, CXCL10) [42] Clearance of fungus in the spleen and lung M1 differentiation Survival Phagocytosis of macrophages and DCs Killing ability of immune cells	Production of cytokines (IL-4, IL-5, IL-6, IL-13, G-CSF) Production of chemokines (CCL2, CCL3, CCL11) [40] Infiltration of PMNs M2 differentiation	Production of IFN-γ Infiltration of leukocytes Clearance of fungus in the brain	[40,41,42]
*E. spinifera*	H (c.68C>A) (c.819- 820insG) (c.191–192insTGCT)	Production of cytokines (IL-1β, IL-6, IL-17, IL-22, IFN-γ, TNF-α) Production of chemokines (CXCL1, CXCL2, CXCL8) Th cells response (Th17, Th22) Activation of NF-κB		ROS production of PBMCs Phagocytosis of PBMCs Th1 cell response	[28]
	M (C57BL/6 vs. CARD9^−/−^)	Production of cytokines (IL-1β, IL-6, IL-17A, IL-22, IFN-γ, TNF-α) Production of chemokines (CXCL1, CXCL2) Infiltration of PMNs Clearance of fungus in footpad Activation of NF-κB and p38		Expression of p38	[28]
*M. irregularis*	M (C57BL/6 vs. CARD9^−/−^)	Production of cytokines (IL-1β, IL-6, IL-10, IL-17A, IL-17p70, IL-23, IFN-γ, TNF-α) Production of chemokines (CXCL1, CXCL2) Activation of NF-κB NETosis Th cells response (Th1, Th17) Clearance of fungus on footpad and lymph node	Production of cytokine (IL-4) Infiltration of immune cells		[49]
*P. murina*	M (C57BL/6 vs. CARD9^−/−^)	Production of cytokines (IL-1β, IL-6, IL-12, TNF-α, G-CSF) in BMDMs Activation of MAPKs and NF-κB Killing ability of macrophages MPO production in the lung Infiltration of immune cells Clearance of fungus in the lung M1 and M2 differentiation Expression of CLRs	Production of cytokine (IFN-γ)	Production of cytokines (IL-4, IL-17) Survival	[51]

* H: human; M: mouse.

CARD9 mediates distinct tissue-specific responses in infections caused by *C. albicans, C. parapsilosis*, and *C. neoformans*. In *C. albicans* infection, CARD9-deficient patients exhibit reduced production of cytokines (IL-1β, IL-6, IL-17, IL-22, TNF-α, GM-CSF, and IFN-γ) and chemokines (CXCL1, CXCL2, and CXCL8) in PBMCs. However, in CARD9-deficient mice, there is an increase in IL-1α, IL-1β, IL-6, CCL2, and TNF-α levels in the kidneys [20,28,29]. Additionally, TNF-α production is decreased in BMDMs but increased in the kidneys of CARD9-deficient mice during *C. albicans* infection. Similarly, in *C. parapsilosis* infection, TNF-α production is increased in BMDMs of CARD9-deficient mice but show no alteration in the kidneys [30]. These findings underscore the differential involvement of CARD9 in immune response to various *Candida* species, although the cytokine production in both *C. albicans* and *C. parapsilosis* infections is regulated by the CARD9/NF-κB signaling [30]. However, the elevated cytokine levels observed in the kidneys of CARD9-deficient mice might suggest the involvement of PRRs other than dectin-1 or signaling pathways unrelated to CARD9.

## 3. Effects of CARD9 on the Immune Response to Bacterial Infections

CARD9 is involved in immune signaling linked to bacterial infections through TLRs (TLR2 and TLR4) and NLRs (NOD1 and NOD2) [5]. Additionally, CLR signaling contributes to antibacterial immunity [52,53,54]. NODs collaborate with CARD9 to facilitate the recognition of bacterial peptidoglycan monosaccharide units known as muramyl dipeptide (MDP). This interaction triggers the activation of the c-Jun N-terminal kinase (JNK) and p38 MAPK signaling pathways, ultimately regulating the production of inflammatory cytokines against bacterial infection [17]. The impact of CARD9 on the immune response to bacterial infections is summarized in Table 2.

### 3.1. Citrobacter rodentium

*Citrobacter rodentium* (*C. rodentium*) is an extracellular intestinal murine-specific pathogen that is commonly used to monitor human pathogenic *Escherichia coli* and inflammatory bowel disease infections [55]. CARD9-deficient mice infected with *C. rodentium* showed increased susceptibility, with increased fecal load and decreased body weight compared to the wild type [56]. Moreover, the expressions of RegIIIγ (an antimicrobial peptide produced by the intestinal Paneth cells), IL-6, and Th17 cytokines (IL-17A and IL-22) were lower compared to the wild type [57]. In addition, they had significantly increased levels of *C. rodentium* in the spleen after infection but showed no difference in survival rates. However, when exposed to a tenfold higher dose of *C. rodentium,* mortality increased in CARD9-deficient mice [57]. TLRs and myeloid differentiation primary response 88 (MYD88) protect the host against *C. rodentium* infection by inducing multiple immune responses, including recruiting neutrophils, macrophages, and DCs and triggering iNOS expression and proliferation of epithelial cells [58,59]. NOD1 and NOD2 induce the host’s innate immune response to *C. rodentium* infection [55]. CARD9 interacts with TLRs and MYD88 or NOD1 and NOD2 to activate MAPKs and NF-κB [12,60,61]. The impaired immune response to *C. rodentium* infection may be a result of the absence of CARD9 in these pathways.

### 3.2. Mycobacterium tuberculosis

*Mycobacterium* is a genus of bacteria that includes several species that can cause a variety of infections in humans and animals. *Mycobacterium tuberculosis* (*M. tuberculosis*) is characterized by its unique cell wall structure, which includes a lipid-rich outer layer that makes it resistant to many antibiotics and disinfectants. *M. tuberculosis* is recognized by a variety of PRRs, including TLRs, complement receptors, scavenger receptors, NLRs, and CLRs [62,63,64].

CARD9-deficient mice infected with *M. tuberculosis* exhibited higher mortality, high bacterial burden, and increased cell death in the lungs, but showed no difference in the spleen [65]. BMDMs from CARD9-deficient mice showed similar levels of nitric oxide (NO) release, internalization, and killing ability similar to the wild type upon *M. tuberculosis* stimulation, but showed impaired production of IL-1β, IL-6, IL-12p40, TNF-α, and CCL5 [65]. Lung homogenates and serum from CARD9-deficient mice showed higher levels of G-CSF, CXCL1, and CCL2 than the wild type. These are cytokines involved in the differentiation and recruitment of neutrophils [65]. As a result, an accumulation of neutrophils and an increase in MPO production were observed in the lungs of CARD9-deficient mice. Despite the impaired cytokine production by macrophages and DCs, the proportion of T cells and the production of cytokines (IL-2, IL-17, IFN-γ, and TNF) in the lungs and spleen of CARD9-deficient mice appeared to be at normal levels [65]. Thus, in *M. tuberculosis* infection, CARD9 is involved in the innate immune response by macrophages and DCs rather than in the adaptive immunity by T cells.

In conclusion, *M. tuberculosis* infection can be recognized by multiple PRRs. CARD9 is involved in cytokine and chemokine secretion during *M. tuberculosis* infections. However, it does not significantly affect T cell function. Furthermore, the high mortality and lung damage in *M. tuberculosis*-infected CARD9-deficient mice can be explained by an excessive immune response with a large increase in neutrophil accumulation.

### 3.3. Staphylococcus aureus

*Staphylococcus aureus* (*S. aureus*) is a bacterium that is commonly found on the skin and mucous membranes of humans and animals. While it is typically a harmless commensal bacterium, it can also be a significant pathogen responsible for a wide range of infections. *S. aureus* is known for its ability to cause skin and soft tissue infections, as well as more serious invasive infections in various parts of the body [66].

PRRs such as TLRs, CLRs, and NLRs are involved in *S. aureus* recognition [67]. However, in an *S. aureus* infection, CARD9 was not involved in the accumulation of neutrophils and organ damage, and its role in the production of cytokines remains controversial [20,29,31,34]. PBMCs from CARD9-deficient patients showed a significant decrease in the production of cytokines IL-1β and IL-6 in response to unopsonized *S. aureus* infection, with no difference in the response to opsonized *S. aureus* [29]. Also, there was no difference in the neutrophils, in both opsonized and unopsonized *S. aureus* infection [29]. However, when PBMCs from CARD9-deficient patients were stimulated with live *S. aureus*, the expression of these cytokines was similar to that of the healthy controls [20]. The brains and spleens of CARD9-deficient mice infected with *S. aureus* showed similar levels of bacterial burden and neutrophil accumulation similar to the wild type [31,34].

Taken together, CARD9-deficient mice showed no impairment in the bacterial burden and neutrophil accumulation in the brain and spleen, suggesting that CARD9 is not involved in fungal clearance and neutrophil accumulation in mice during an *S. aureus* infection. Reports of conflicting cytokine biology during *S. aureus* infections in CARD9-deficient patients may be due to mutational differences between patients or differences in the experimental procedures. For example, the cytokine production from the PBMCs of patients with CARD9 mutation (c.883C>T) was normal following *S*. *aureus* stimulation [20]. However, it was impaired in another patient with a different mutation (c.214G>A and c.1118G>C) [29].

### 3.4. Salmonella enterica serovar Typhimurium

*Salmonella* Typhimurium (*S.* Typhimurium) is a strain of bacteria of the genus *Salmonella* that is known to cause a variety of foodborne illnesses in humans and animals. *S.* Typhimurium is one of the most common *Salmonella* serotypes associated with human infections [68].

Intracellular *Salmonella* activates NLRs that can induce IL-23 expression and assembles the NLR family CARD domain-containing 4 (NLRC4)/NLR family pyrin domain-containing protein 3 (NLRP3) inflammasomes that activate caspase-1 to promote the secretion of mature IL-1β and IL-18 [69]. BMDMs from CARD9-deficient mice showed an increase in IL-1β and a decrease in pyroptosis in response to *S.* Typhimurium infections, indicating that CARD9 negatively regulates IL-1β production in the inflammasome [70]. This response contrasts with the role of CARD9 in inducing an infectious response to fungal infections. Increased IL-1β is caused by enhanced NLRP3 activation after *S.* Typhimurium infection, and CARD9 inhibits Syk phosphorylation [70]. In this process, CARD9 regulates the innate immune inflammatory response, acting as a negative regulator of IL-1β production in macrophages by regulating pro-IL-1β expression and caspase-8 mobilization to the inflammasome [70].

### 3.5. Streptococcus pneumoniae

*Streptococcus pneumoniae* (*S. pneumoniae*)*,* commonly referred to as pneumococcus, is a bacterium that lives in the human upper respiratory tract, especially in the nose and throat. While it is a commensal bacterium in healthy individuals, it is also an important human pathogen that causes a variety of infections, including invasive diseases such as pneumonia, otitis media, sinusitis, bacteremia, and meningitis [71].

CARD9-deficient mice showed reduced specific-immune cell infiltration in the lungs compared to the wild type in response to pneumococcal infection; specifically, the number of neutrophils was significantly reduced compared to the wild type, but the number of macrophages was not altered [54]. This is because the cytokines and chemokines involved in neutrophil recruitment are reduced by CARD9 deficiency [54]. The alveolar macrophages showed a CARD9-deficiency-induced decrease in cytokines (TNF-α and CXCL1), in particular, CXCL1 via mincle [54], suggesting that neutrophil recruitment via mincle in *S. pneumoniae* infection occurs in a CARD9-dependent manner. The defects, such as immune cell infiltration and cytokine production seen in these CARD9-deficient mice, were not seen in dectin-2-deficient mice, which showed defects in phagocytosis of neutrophils [54]. In summary, the phagocytosis of neutrophils during pneumococcal infection occurs through a dectin-2-CARD9-dependent mechanism, but responses such as neutrophil recruitment and macrophage production of TNF-α, CXCL1, and CXCL2 are mediated by the signaling pathways of other CLRs, not dectin-2.

**Table 2:** Role of CARD9 in bacterial infections.

Pathogens: Bacteria	Host	Effects of CARD9			Refs.
		Positive	Negative	No Effect	
*C. rodentium*	M * (C57BL/6 vs. CARD9^−/−^)	Production of cytokines (IL-6, IL-17A, IL-22, RegIIIγ) Survival Bacterial susceptibility			[56,57]
*M. tuberculosis*	M (C57BL/6 vs. CARD9^−/−^)	Production of cytokines (IL-1β, IL-6, IL-12p40, TNF) Production of chemokine (CCL5) Survival Clearance of bacterium in the lung	Production of cytokines (CXCL1, CCL2, G-CSF)		[65]
*S. aureus*	H * (c.883C>T) (c.214G>A) (c.1118G>C)	Production of cytokines (IL-1β, IL-6) [29]		Production of cytokines (IL-1β, IL-6) [20] and (IL-8) [29]	[20,29]
	M (C57BL/6 vs. CARD9^−/−^)			Infiltration of PMNs Clearance of bacterium in the brain and spleen	[31,34]
*S.* Typhimurium	M (C57BL/6 vs. CARD9^−/−^)	Pyroptosis in BMDMs	Production of cytokine (IL-1β) Activation of Syk		[69,70]
*S. pneumoniae*	M (C57BL/6 vs. CARD9^−/−^)	Production of cytokines (IL-12p40, IFN-γ, TNF-α) Production of chemokines (CXCL1, CXCL2) Phagocytosis of PMNs Infiltration of PMNs Clearance of bacterium in the lung		Infiltration of macrophages	[54]

* H: human; M: mouse.

## 4. Effects of CARD9 on the Immune Response to Viral Infections

Several PRRs are involved in the recognition of viral components, and CARD9 plays a crucial function in viral infections [17]. CARD9 interacts with viral DNA or RNA, triggering the activation of NF-κB [72,73]. Studies investigating the significance of CARD9 with respect to the immune response to viral infections are compiled in Table 3.

### 4.1. Coxsackievirus B3

Coxsackievirus B3 (CVB3) is a virus of the genus *Enterovirus* that infects multiple organs, sometimes causing severe systemic disease, including myocarditis and pancreatitis [74]. During CVB3 infection, CARD9-deficient mice expressed lower levels of BCL10, TGF-β, and IL-17, along with a diminished production of cytokines (IL-6, IL-10, IL-17A, IFN-γ, and TGF-β) compared to wild-type mice [75]. However, the expression level of Syk was similar to that of the wild type. This suggests that CARD9 interacts with BCL10 through Syk-independent signaling to induce an inflammatory response in CVB3 infection. Furthermore, CARD9-deficient mice exhibited a lower pathological score during CVB3 infection compared to the wild type, which also indicates Syk-independent signaling [75].

Taken together, CARD9 induces the production of cytokines (IL-6, IL-10, IL-17A, IFN-γ, and TGF-β) through the Syk-independent BCL10 signaling pathway in CVB3 infections, and these cytokines are involved in the induction and differentiation of regulatory T cells and Th17 cells [76,77]. Therefore, targeting CARD9 may offer a new therapeutic approach to CVB3 infections, such as viral myocarditis.

### 4.2. Influenza Virus

Influenza viruses (IFVs) are members of the family *Orthomyxoviridae*, which contain a negative-sense, single-stranded, segmented RNA genome protected by the capsid of the viral ribonucleoprotein. It is classified into subtypes based on the expression of hemagglutinin and neuraminidase on the surface of the viral envelope [78].

Influenza pneumonia was dramatically attenuated in CARD9-deficient mice, which showed improved mortality with reduced inflammatory cytokines and chemokines in the infected lungs [79]. During IFV infections in CARD9-deficient mice, there was reduced infiltration of T cells and neutrophils. However, the infiltration of B cells, NK cells, macrophages, and DCs remained intact [79]. Upon IFV infection, the levels of cytokines (IL-6 and TNF-α) and chemokines (CCL3, CXCL1, and interferon gamma-induced protein (IP)-10) were reduced in the lungs of CARD9-deficient mice which recovered with time [79]. This suggests that CARD9 is involved in early cytokine and chemokine production in IFV infections. Moreover, the production of cytokines and chemokines in the lungs was impaired, but IFN-α/β/γ production, CD8 T cell development, and IgG and IgA production were not altered [79]. In addition, during IFV infections, impairment in the production of IL-6 and TNF-α was observed in DCs but not in macrophages [79]. Inhibition of Syk in DCs reduces IL-6 and TNF-α, but not IFN-α/β production [79]. Thus, the Syk/CARD9 signaling pathway probably controls the production of cytokines in DCs. Overall, CARD9 is involved in cytokine (IL-6 and TNF-α) production by DCs and the infiltration of T cells and neutrophils but does not alter viral burden, the elevation of IFN-α/β, or the induction of antiviral adaptive T and B cell responses in the IFV-infected mice.

### 4.3. La Crosse Virus

La Crosse virus is the most pathogenic member of the California encephalitis serogroup and is the leading cause of neuroinvasive viral disease in young children, accounting for up to 55% of all reported cases [80,81]. PRRs such as RIRs, TLRs, and CLRs are involved in the recognition of the La Crosse virus [82,83,84,85]. Deficiency of mincle or CARD9 reduced the production of IL-6 and TNF-α but does not affect viral clearance [85]. Thus, the mincle/CARD9 signaling pathway is involved in La Crosse virus recognition and cytokine production but plays a limited role in viral clearance.

### 4.4. Theiler’s Murine Encephalomyelitis Virus

Theiler’s murine encephalomyelitis virus (TMEV) is a neurotropic picornavirus that temporally affects the hippocampus and damages nerve cells [86,87]. CARD9-deficient mice showed an increased transient viral burden in the brain, hippocampal damage, and impaired production of cytokines (IL-1β and IFN-γ) during TMEV infections [88]. However, IL-5 tended to increase, which may be a compensatory response to CARD9 deficiency. CARD9-deficient mice showed increased M2 macrophages and T cell counts during TMEV infection [88]. However, the ability to clear viruses and produce cytokines (IL-1α, IL-4, IL-6, IL-10, TGF-β1, and TNF-α) and prime T cells was not impaired by the CARD9 deficiency. Taken together, CARD9 can prevent hippocampal damage during TMEV infections by regulating IL-1β and IFN-γ production and T cell count but is not required for viral clearance.

**Table 3:** Role of CARD9 in viral infections.

Pathogen: Viruses	Host	Effects of CARD9			Refs.
		Positive	Negative	No Effect	
Coxsackievirus B3	M * (C57BL/6 vs. CARD9^−/−^)	Production of cytokines (IL-6, IL-10, IL-17A, IFN-γ, TGF-β) Expression of BCL10		Expression of Syk Viral burden	[75]
Influenza virus	M (C57BL/6 vs. CARD9^−/−^)	Production of cytokines (IL-6, TNF-α) in the lung and DCs Production of chemokines (CXCL1, CCL3, IP-10) Infiltration of T cells and PMNs	Production of cytokine (IFN-γ) in the lung Survival Clearance of virus	Production of cytokines (IL-6, IFN-α/β, TNF-α) in macrophages Infiltration of B cells, NK cells, macrophages, and DCs Production of IgA and IgG	[79]
La Crosse virus	M (C57BL/6 vs. CARD9^−/−^)	Production of cytokines (IL-6, TNF-α)		Clearance of virus	[85]
Theiler’s murine encephalomyelitis virus	M (C57BL/6 vs. CARD9^−/−^)	Production of cytokines (IL-1β, IFN-γ) Hippocampal damage	Production of cytokine (IL-5) M2 differentiation T cells infiltration	Production of cytokines (IL-1α, IL-4, IL-6, IL-10, TNF-α, TGF-β1) Clearance of virus T cells priming	[88]

* M: mouse.

## 5. Effects of CARD9 on the Immune Response against Miscellaneous Stimuli

### 5.1. CLR Ligands (Curdlan, Mannan, and TDB)

Curdlan is a linear, β-1,3-glucan polysaccharide produced by bacteria, particularly by the *Agrobacterium* species, *Bacillus* species, and *Cellulomonas* species. It is similar to fungal cell wall component that has been identified as a dectin-1-specific ligand [89,90]. PBMCs from CARD9-deficient patients produce less IL-1β, IL-6, IL-22, IFN-γ, and TNF-α and fewer Th1, Th17, and Th22 cells in response to curdlan stimulation compared to healthy controls [21].

Mannan is a type of polysaccharide comprising of mannose sugar units. It is found in various natural sources, including the cell walls of yeast and certain plant tissues. Mannan acts as a dectin-2-specific ligand [43,52]. PBMCs from CARD9-deficient patients showed a decrease in cytokines (IL-1β, IL-6, IL-17A, IL-22, IFN-γ, and TNF-α) upon mannan stimulation, with fewer Th1, Th17, and Th22 cells [21]. BMDMs from CARD9-deficient mice also showed significantly lower levels of IL-6, IL-10, IL-12p40, and TNF production than the wild type upon mannan stimulation [43].

Trehalose-6,6-dibehenate (TDB) is a glycolipid found in the cell walls of mycobacteria, including *M. tuberculosis*. TDB acts as a mincle-specific ligand and induces Th1 and Th17 immune responses [91,92]. It also activates the CARD9 signaling pathway in myeloid cells [93]. Stimulation of PBMCs from CARD9-deficient patients with TDB significantly reduced cytokines (IL-1β, IL-6, IL-17A, IL-22, IFN-γ, and TNF-α) and Th cells (Th1, Th17, and Th22) compared to the wild type [21]. Consistent with this, BMDCs from CARD9-deficient mice also showed defects in TNF-α and IL-1β production in response to TDB stimulation [94].

### 5.2. Zymosan

Zymosan is a type of molecule derived from the cell walls of the yeasts, typically the cell walls of *Saccharomyces cerevisiae*. It is often used in laboratory research to study the activation of the innate immune system and as a tool for simulating microbial infections. Zymosan is a ligand for TLRs and dectin-1 which together trigger an inflammatory response [95]. In contrast, depleted zymosan that was obtained by treating zymosan with hot alkali to remove its TLR-stimulating properties can only signal through dectin-1 [95,96].

Macrophages derived from CARD9-deficient mice exhibited lower TNF-α levels compared to the wild type in response to zymosan stimulation, and neither wild-type nor CARD9-deficient macrophages produced TNF-α in response to depleted zymosan that had been prepared by boiling zymosan in sodium hydroxide [97]. The IL-2, IL-6, and TNF-α production by zymosan stimulation and TNF-α level by depleted zymosan stimulation were reduced in CARD9-deficient DCs [34,97]. Consistent with this, zymosan and depleted zymosan induced NF-κB activation in DCs, but depleted zymosan failed to induce NF-κB activation in macrophages [97].

Despite decreased TNF-α production and reduced NF-κB activation due to CARD9 deficiency, phagocytosis of both macrophages and DCs to zymosan stimulation was unimpaired [97]. The neutrophils from CARD9-deficient patients showed normal levels of hydrogen peroxide (H_2_O_2_) production upon zymosan stimulation [29,97]. CARD9-deficient mice showed increased IL-1β and CCL2 during zymosan infection, with macrophages showing increased M1 polarization, decreased M2 polarization, and increased infiltration into the site of infection, whereas neutrophils and T cells showed no difference in infiltration [98]. Despite the increase in inflammatory cell infiltration, CARD9-deficient mice exhibited a higher susceptibility to zymosan-induced peritonitis compared to the wild type, which may have resulted from the impaired autophagy of macrophages [98].

Thus, we can see that CARD9 is involved in the zymosan-induced immune response by participating in the polarization and autophagy of macrophages. However, CARD9 is not involved in phagocytosis after zymosan stimulation in macrophages and DCs.

### 5.3. TLR Ligands (LPS and Pam3CSK4)

Lipopolysaccharide (LPS) is an outer membrane component of Gram-negative bacteria, and Pam3CysSerLyn4 (Pam3CSK4) is a synthetic triacylated lipopeptide. They are both molecules that play significant roles in the immune system and are often used in research to study immune responses [99,100].

LPS is recognized by TLR4 and induces the production of pro-inflammatory cytokines [101]. Pam3CSK4 is known as a TLR2-specific ligand [102]. Conflicting results have been reported on the production of cytokines (IL-1β, IL-6, and TNF-α) in PBMCs from CARD9-deficient patients and mice in response to LPS stimulation [10,20,21,28]. PBMCs from patients and mice with CARD9 deficiency produced similar levels of IL-1β, IL-6, and TNF-α compared to PBMCs from healthy individuals upon LPS stimulation [10,20,28]. However, other studies have reported reduced levels of IL-1β and TNF-α compared to control PBMCs [21]. These conflicting reports may be due to mutations in patients or differences in the experimental conditions used in the studies. However, there is a preponderance of evidence that these cytokines are unaffected by LPS stimulation [20,28,34]. In mice, CARD9-deficient macrophages exhibited NF-κB activation comparable to that of the wild type in response to LPS stimulation, while nitric oxide release was reduced [10,30,40]. Taken together, these data suggest that CARD9 does not significantly participate in cytokine production in the Pam3CSK4- or the LPS-induced TLR2/4 signaling pathway but is involved in the oxidative stress of macrophages in mice. Macrophages and DCs from CARD9-deficient mice produced similar levels of TNF-α to the wild type in response to Pam_3_CSK_4_ [97].

### 5.4. MDP

MDP is a bacterial peptidoglycan monosaccharide that is composed of two amino acids, L-alanine and D-glutamine, linked to a sugar, N-acetyl-muramic acid. It is found in the cell walls of Gram-positive and Gram-negative bacteria. It is a representative small-molecule peptidoglycan mimic of bacteria and has been shown to interact with NLRs, such as NOD2 and NLRP1 [103]. PBMCs from CARD9-deficient patients were impaired and cytokines (IL-1β, IL-6, and TNF-α) were decreased in response to MDP [28], suggesting that CARD9 is involved in the host immune response in the NOD2 induction pathway.

### 5.5. Dextran Sulfate Sodium

Dextran sulfate sodium (DSS) is a negatively charged sulfated polysaccharide that is often used in laboratory research to induce colitis in animal models [104]. CARD9-deficient mice stimulated with DSS have reduced body weight and a shorter colon length compared to the wild type [57,105,106], and show impaired production of cytokines (IL-6, IL-17A, IL-21, IL-22, IL-23, and IFN-γ) and CCL20 in the colon of CARD9-deficient mice [58,105]. Additionally, TNF-α and IFN-γ were decreased in CARD9-deficient T cells [106]. Moreover, in DSS-induced colitis, the absence of CARD9 in neutrophils increases mitochondrial ROS production leading to apoptosis, especially in oxidative environment [32,107].

### 5.6. Hydrogen Peroxide

Hydrogen peroxide plays a role as a signaling molecule and is involved in various physiological processes [108]. The expression of cleaved caspase 3 and 9, and the number of apoptotic cells were increased in cardiomyocytes and neutrophils of CARD9-deficient mice [32,107], suggesting that CARD9 is involved in apoptosis in response to H_2_O_2_ stimulation in neutrophils and cardiomyocytes.

### 5.7. Particulate Matter

Particulate matter (PM) refers to tiny particles or droplets in the air that can be composed of various materials, including dust, soot, smoke, liquid droplets, and solid particles. These particles can vary in size, composition, and origin and have significant implications for air quality, human health, and the environment [109]. Upon stimulation by PM, CARD9-deficient mice exhibited reduced ROS production and macrophage infiltration compared to the wild type [110]. PM stimulation significantly increased M1 polarization and suppressed M2 polarization via the ROS- and mammalian target of rapamycin (mTOR)-mediated pathways, respectively [111]. These findings suggest that CARD9-mediated signaling is essential to produce ROS and the induction of inflammatory responses in macrophages. The effects of CARD9 in response to various stimuli are summarized in Table 4.

**Table 4:** Role of CARD9 in response to various stimuli.

Stimuli	Host	Effects of CARD9			Refs.
		Positive	Negative	No Effect	
Curdlan, TDB, and Mannan	H * (c.819-820insG)	Production of cytokines (IL-1β, IL-6, IL-10, IL-12p40, IL-17A, IL-22, IFN-γ, TNF-α) Th cells response (Th1, Th17, Th22)			[21]
	M * (C57BL/6 vs. CARD9^−/−^) RAW 264.7 cell	Production of cytokines (IL-1β, IL-6, IL-17A, IL-22, IFN-γ, TNF-α) Activation of NF-κB (mannan)		Activation of NF-κB (curdlan)	[43,94,112]
Dextran sulfate sodium	M (C57BL/6 vs. CARD9^−/−^)	Production of cytokines (IL-6, IL-17A, IL-22, IFN-γ, TNF-α) Production of chemokines (CCL2, CCL20) Bodyweight and colon length	Production of MPO Infiltration of monocytes, macrophages, and PMNs [106]	Infiltration of B and T cells [106] and PMNs [32]	[32,57,105,106]
Hydrogen peroxide	M (C57BL/6 vs. CARD9^−/−^) H9c2 cells		Apoptosis of cardiomyocytes and PMNs	Cytochrome C release	[32,107]
LPS and Pam3CSK4	H (c.3G>C) (c.819_820insG) (c.68C>A)	Production of cytokines (IL-1β, TNF-α) [21]		Production of cytokines (IL-1β, IL-6, TNF-α, GM-CSF) [20,28]	[20,21,28]
	M (C57BL/6 vs. CARD9^−/−^)	Production of NO in macrophages		Production of cytokines (IL-6, TNF-α) [10] Activation of NF-κB and MAPKs	[10,30,40,97]
MDP	H (c.68C>A) (c.819-820insG)	Production of cytokines (IL-1β, IL-6, TNF-α)			[28]
Particulate matter	M (C57BL/6 vs. CARD9^−/−^)	ROS production of macrophages Infiltration of inflammatory cells			[110,111]
depleted Zymosan	M (C57BL/6 vs. CARD9^−/−^)	Production of cytokines (IL-2, IL-6, TNF-α) in DCs			[97]
Zymosan	H (c.214G>A) (c.1118G>C)			Production of H_2_O_2_	[29,97]
	M (C57BL/6 vs. CARD9^−/−^)	Production of cytokine (IL-2, IL-6, TNF-α) in DCs Production of cytokine (TNF-α) in macrophages Survival Autophagy of macrophages M2 differentiation	Production of cytokine (IL-1β) Production of chemokine (CCL2) Infiltration of macrophages M1 differentiation	Phagocytosis of macrophages and DCs Infiltration of T cells and PMNs	[34,97,98]

* H: human; M: mouse.

## 6. Conclusions

Activation of the immune response during infections is essential for pathogen clearance and effective host defense. Innate immune cells play an important role in pathogen clearance early in an infection. CARD9 is involved in the recruitment of innate immune cells like neutrophils, macrophages, and DCs. CARD9 plays a critical role in responding to pathogens, and its functions differ depending on the cell and tissue types as well as the nature of the pathogen. The distinctions among various infections concerning primary effector cells and functions are summarized in Table 5.

In the case of fungal infections, CARD9 primarily regulates cytokine and chemokine production and cell migration. A CARD9 deficiency results in a decrease in inflammatory cytokines (IL-1β, IL-6, and TNF-α) and chemokines (CXCL1, CXCL2, CXCL4, and CXCL5) in both humans and mice [20,21,22,23,28,29,30,31,32,33,34,37,38,40,42,49,51]. CARD9 deficiency results in a reduced infiltration of innate immune cells, and this may result from a decrease in chemokines, which play a role in recruiting immune cells. However, discrepancies have been noted between *C. albicans* and *C. parapsilosis* infection [30]. CARD9-deficient cells revealed no impairment in phagocytosis and killing ability during *A. fumigatus* [20] and certain *Candida* species infections [30,98]. However, in other studies, a CARD9 deficiency resulted in impaired phagocytosis and killing ability against unopsonized *C. albicans*, *C. neoformans*, and *Pneumocystis* infections [29,32,40,41,51]. These conflicting observations suggest that CARD9 plays a pathogen- and cell-specific role in the infiltration, phagocytosis, and killing by innate immune cells.

In bacterial infections, a CARD9 deficiency results in a decrease in inflammatory cytokines (IL-1β, IL-6, and TNF-α) and chemokines (CXCL1, CXCL2, and CXCL5) [20,21,22,23,28,29,30,31,32,33,34,37,38,40,42,49,51]. Survival was reduced in CARD9-deficient mice infected with *C. rodentium* and *M. tuberculosis* [20,21,22,23,28,29,30,31,32,33,34,37,38,40,42,49,51], and the clearance of bacteria was impaired in *M. tuberculosis* or *S. pneumoniae* infections [20,21,22,23,28,29,30,31,32,33,34,37,38,40,42,49,51]. In addition, CARD9 plays various roles in viral infections. It contributes to cytokine production and specific immune cell infiltration in CVB3 and IFV infections, influencing the immune response, whereas in La Crosse virus infection, it participates in cytokine production without significantly affecting viral clearance [20,21,22,23,28,29,30,31,32,33,34,37,38,40,42,49,51]. The primary cells and main immunological functions regulated by CARD9 are summarized in Table 5.

**Table 5:** The primary cells and immunological functions that CARD9 regulates.

Species	Pathogens	Primary Cells	Functions	Refs.
Fungi	*A. fumiagtus*	PMNs PBMCs	Production of cytokines Infiltration Phagocytosis Killing ability	[20,21,22,23]
	*C. albicans*	PMNs PBMCs	Production of cytokines Infiltration Phagocytosis Killing ability ROS production	[20,27,28,29,30,31,32]
	*C. parapsilosis*	BMDMs	Production of cytokines Phagocytosis	[30]
	*C. tropicalis*	PMNs BMDMs Monocytes	Killing ability	[37,38]
	*C. neoformans*	PMNs BMDMs BMDCs	Infiltration Phagocytosis Anticryptococcal activity	[40,41,42]
	*E. spinifera*	PBMCs BMDMs	Production of cytokines Phagocytosis ROS production Activation of NF-κB and MAPKs	[28]
	*M. irregularis*	PMNs BMDMs BMDCs	Production of cytokines NETosis Activation of NF-κB	[49]
	*P. murina*	PMNs BMDMs	Production of cytokines Infiltration Activation of MAPKs	[51]
Bacteria	*C. rodentium*	PMNs Macrophages DCs	Infiltration	[58,59]
	*M. tuberculosis*	BMDMs PMNs	Production of cytokines Production of NO Infiltration Internalization Killing ability MPO production	[65]
	*S. aureus*	PMNs PBMC	Production of cytokines Infiltration	[20,29,31,34]
	*S.* Typhimurium	BMDMs	Production of cytokines Pyroptosis	[70]
	*S. pneumoniae*	PMNs Macrophages	Production of cytokines Infiltration Phagocytosis	[54]
Virus	Influenza virus	PMNs Macrophages DCs	Production of cytokines Infiltration	[79]
	Theiler’s murine encephalomyelitis virus	Macrophages	Differentiation	[88]

Collectively, CARD9 predominantly governs the migration of inflammatory cells and the production of cytokines. Some cytokines are directly produced downstream of CARD9 through the regulation of NF-κB and others are likely to be induced by the cytokines regulated by CARD9 (secondary effects). CARD9 can indeed synergize with other signals, particularly those activating NF-κB like TNF or IL-18. The nature and impact of this synergistic interplay on various infections may vary. In certain infections, it can enhance pro-inflammatory cytokine production and promote phagocyte activation, contributing to pathogen clearance. On the other hand, in certain inflammatory conditions, excessive activation of TNF and IL-18 may result in tissue damage and exacerbate inflammation.

During infections, CARD9-deficient humans and mice have an increased pathogen burden. This results from impairments in the infiltration of immune cells or the clearance of pathogens, such as phagocytosis. However, some organs are unaffected by CARD9 deficiency during infection by certain pathogens. For instance, the *C. tropicalis* infection resulted in an increased fungal burden in the kidney, brain, and liver of CARD9-deficient mice, but there was no increased fungal burden in the spleen [37]. This response is different from the response to infection by other members of the *Candida* species, such as *C. albicans* or *C. parapsilosis*. Similarly, a *C. neoformans* infection increases the fungal burden in the spleen and lungs of CARD9-deficient mice, but not in the brain [40]. In bacterial infections caused by *S. aureus*, *S. pneumoniae*, or *Mycobacterium*, different organs are affected [31,34,54,65]. This suggests that the role of CARD9 varies depending on the type of pathogen and the organ is involved in the immune response. To date, we cannot explain why the CARD9 deficiency plays a context-dependent role in different infections, even with the same type of pathogen. However, this phenomenon may reflect the multifaceted nature of the immune responses and the intricate interplay between pathogen variations, immune cell types, immune crosstalk, and the inflammatory context. Understanding these complexities is essential for elucidating the mechanisms underlying CARD9-mediated functions and developing targeted therapeutic interventions.

CARD9 also plays a crucial role in orchestrating Th cell-mediated immune responses. Decreased proportions of Th cells (Th1, Th2, and Th17) and Th cell responses are observed in CARD9 deficiency. In both CARD9-deficient humans and mice, decreased levels of Th1 and Th17 cytokines are evident in response to numerous fungal stimuli [20,21,28,40]. Conversely, the levels of Th2 cytokines remain normal or sometimes even elevated [28,30,40,49,51]. This suggests that CARD9 influences Th1- and Th17-related responses but has a less pronounced role in Th2-related responses. Similarly, in immune responses to bacterial infection and CLR ligands, a CARD9 deficiency leads to reductions in Th1 and Th17 cytokines [21,43,54,56,57,70,94]. Furthermore, during fungal infections, such as *A. fumigatus*, *C. albicans*, *E. spinifera*, and *M. irregularis*, CARD9 plays a regulatory role in immune cell polarization and differentiation. A CARD9 deficiency results in decreased polarization of macrophages into M1 and M2, although these reductions are diverse and fungus specific.

In summary, CARD9 contributes to the host defense by participating in diverse signaling events essential for the immune response against various pathogens. Its involvement spans the regulation of cytokine and chemokine production, cell migration, phagocytosis, Th cell-mediated immune responses, and the polarization and differentiation of macrophages. CARD9 elicits cell- and organ-specific immune responses depending on the pathogen types. Therefore, understanding the intricate interactions between pathogens and cells is a crucial factor in grasping the pivotal role of CARD9 in host defense responses.

## Data Availability

Not applicable.

## Abbreviations

ALRs	AIM2-like receptors
BCL	B-cell lymphoma/leukemia
BMDCs	Bone marrow-derived dendritic cells
BMDMs	Bone marrow-derived macrophages
CARD9	Caspase recruitment domain-containing protein 9
CBM	CARD9-BCL10-MALT1
CLRs	C-type lectin receptors
CNS	Central nervous system
DC-SIGN	Dendritic cell-specific ICAM-grabbing non-integrin
DCs	Dendritic cells
DSS	Dextran sulfate sodium
G-CSF	Granulocyte colony-stimulating factor
GM-CSF	Granulocyte-macrophage colony-stimulating factor
ICAM	Intracellular adhesion molecule
IFN	Interferon
IL	Interleukin
IP	Interferon gamma-induced protein
JNK	c-Jun N-terminal kinase
LP	Lipopeptide
LPS	Lipopolysaccharide
MALT	Mucosa-associated lymphoid tissue lymphoma translocation protein
MAPK	Mitogen-activated protein kinase
MDP	Muramyl dipeptide
MIP	Macrophage inflammatory protein
MPO MYD88	Myeloperoxidase Myeloid differentiation primary response 88
NETs	Neutrophil extracellular traps
NF-κB NK NLRC4	Nuclear factor kappa-light-chain-enhancer of activated B cells Natural killer NLR family CARD domain-containing 4
NLPR3	NLR family pyrin domain-containing protein 3
NLRs	NOD-like receptors
NOD	Nucleotide oligomerization domain
OLRs	OAS-like receptors
Pam3CSK4	Pam3CysSerLyn4
PAMPs	Pathogen-associated molecular patterns
PGBPs	Peptidoglycan-binding proteins
PBMCs	Peripheral blood mononuclear cells
PGN	Peptidoglycans
PMNs	Polymorphonuclear leukocytes
PRRs	Pattern recognition receptors
RIG	Retinoic acid-inducible gene
RLRs	RIG-I-like receptors
ROR	Retinoic acid receptor-related orphan receptor
ROS	Reactive oxygen species
SLAMF	Signaling lymphocytic activation molecule family
Syk	Spleen tyrosine kinase
TDB	Trehalose-6,6-dibehenate
TGF	Transforming growth factor
Th	T helper
TLRs	Toll-like receptors
TNF	Tumor necrosis factor

## References

[1] Takeuchi O., Akira S. (2010). Pattern recognition receptors and inflammation. Cell.

[2] Thaiss C.A., Levy M., Itav S., Elinav E. (2016). Integration of innate immune signaling. Trends Immunol..

[3] Chuenchor W., Jin T., Ravilious G., Xiao T.S. (2014). Structures of pattern recognition receptors reveal molecular mechanisms of autoinhibition, ligand recognition and oligomerization. Curr. Opin. Immunol..

[4] Hoving J.C., Wilson G.J., Brown G.D. (2014). Signalling C-type lectin receptors, microbial recognition and immunity. Cell. Microbiol..

[5] Saxena M., Yeretssian G. (2014). NOD-like receptors: Master regulators of inflammation and cancer. Front. Immunol..

[6] Loo Y.M., Gale M. (2011). Immune signaling by RIG-I-like receptors. Immunity.

[7] Ji C., Yang Z., Zhong X., Xia J. (2021). The role and mechanism of CARD9 gene polymorphism in diseases. Biomed. J..

[8] Bertin J., Guo Y., Wang L., Srinivasula S.M., Jacobson M.D., Poyet J.L., Merriam S., Du M.Q., Dyer M.J., Robison K.E. (2000). CARD9 is a novel caspase recruitment domain-containing protein that interacts with BCL10/CLAP and activates NF-kappa B. J. Biol. Chem..

[9] Lupas A. (1996). Coiled coils: New structures and new functions. Trends Biochem. Sci..

[10] Hsu Y.M., Zhang Y., You Y., Wang D., Li H., Duramad O., Qin X.F., Dong C., Lin X. (2007). The adaptor protein CARD9 is required for innate immune responses to intracellular pathogens. Nat. Immunol..

[11] Drummond R.A., Lionakis M.S. (2016). Mechanistic insights into the role of C-type lectin receptor/CARD9 signaling in human antifungal immunity. Front. Cell. Infect. Microbiol..

[12] Wang Y., Zhang D., Hou Y., Shen S., Wang T. (2020). The adaptor protein CARD9, from fungal immunity to tumorigenesis. Am. J. Cancer Res..

[13] Vornholz L., Ruland J. (2020). Physiological and pathological functions of CARD9 signaling in the innate immune system. Curr. Top. Microbiol. Immunol..

[14] Tayal V., Kalra B.S. (2008). Cytokines and anti-cytokines as therapeutics—An update. Eur. J. Pharmacol..

[15] Murphy K.W., Weaver C., Berg L. (2022). Janeway’s Immunobiology.

[16] Turner M.D., Nedjai B., Hurst T., Pennington D.J. (2014). Cytokines and chemokines: At the crossroads of cell signalling and inflammatory disease. Biochim. Biophys. Acta.

[17] Drummond R.A., Franco L.M., Lionakis M.S. (2018). Human CARD9: A critical molecule of fungal immune surveillance. Front. Immunol..

[18] Palomino D.C., Marti L.C. (2015). Chemokines and immunity. Einstein.

[19] van de Veerdonk F.L., Gresnigt M.S., Romani L., Netea M.G., Latge J.P. (2017). Aspergillus fumigatus morphology and dynamic host interactions. Nat. Rev. Microbiol..

[20] Rieber N., Gazendam R.P., Freeman A.F., Hsu A.P., Collar A.L., Sugui J.A., Drummond R.A., Rongkavilit C., Hoffman K., Henderson C. (2016). Extrapulmonary Aspergillus infection in patients with CARD9 deficiency. JCI Insight.

[21] Zhang Y., Huang C., Song Y., Ma Y., Wan Z., Zhu X., Wang X., Li R. (2021). Primary cutaneous Aspergillosis in a patient with CARD9 deficiency and Aspergillus susceptibility of Card9 knockout mice. J. Clin. Immunol..

[22] Jhingran A., Mar K.B., Kumasaka D.K., Knoblaugh S.E., Ngo L.Y., Segal B.H., Iwakura Y., Lowell C.A., Hamerman J.A., Lin X. (2012). Tracing conidial fate and measuring host cell antifungal activity using a reporter of microbial viability in the lung. Cell Rep..

[23] Jhingran A., Kasahara S., Shepardson K.M., Junecko B.A., Heung L.J., Kumasaka D.K., Knoblaugh S.E., Lin X., Kazmierczak B.I., Reinhart T.A. (2015). Compartment-specific and sequential role of MyD88 and CARD9 in chemokine induction and innate defense during respiratory fungal infection. PLoS Pathog..

[24] Sun H., Xu X.Y., Tian X.L., Shao H.T., Wu X.D., Wang Q., Su X., Shi Y. (2014). Activation of NF-kappaB and respiratory burst following Aspergillus fumigatus stimulation of macrophages. Immunobiology.

[25] Zheng N.X., Wang Y., Hu D.D., Yan L., Jiang Y.Y. (2015). The role of pattern recognition receptors in the innate recognition of Candida albicans. Virulence.

[26] Saijo S., Fujikado N., Furuta T., Chung S.H., Kotaki H., Seki K., Sudo K., Akira S., Adachi Y., Ohno N. (2007). Dectin-1 is required for host defense against Pneumocystis carinii but not against Candida albicans. Nat. Immunol..

[27] Drummond R.A., Swamydas M., Oikonomou V., Zhai B., Dambuza I.M., Schaefer B.C., Bohrer A.C., Mayer-Barber K.D., Lira S.A., Iwakura Y. (2019). CARD9(+) microglia promote antifungal immunity via IL-1beta- and CXCL1-mediated neutrophil recruitment. Nat. Immunol..

[28] Wang X., Zhang R., Wu W., Song Y., Wan Z., Han W., Li R. (2018). Impaired specific antifungal immunity in CARD9-deficient patients with phaeohyphomycosis. J. Investig. Dermatol..

[29] Drewniak A., Gazendam R.P., Tool A.T., van Houdt M., Jansen M.H., van Hamme J.L., van Leeuwen E.M., Roos D., Scalais E., de Beaufort C. (2013). Invasive fungal infection and impaired neutrophil killing in human CARD9 deficiency. Blood.

[30] Zajta E., Csonka K., Toth A., Tiszlavicz L., Nemeth T., Orosz A., Novak A., Csikos M., Vagvolgyi C., Mocsai A. (2021). Signaling through Syk or CARD9 mediates species-specific anti-Candida protection in bone marrow chimeric mice. mBio.

[31] Drummond R.A., Collar A.L., Swamydas M., Rodriguez C.A., Lim J.K., Mendez L.M., Fink D.L., Hsu A.P., Zhai B., Karauzum H. (2015). CARD9-dependent neutrophil recruitment protects against fungal invasion of the central nervous system. PLoS Pathog..

[32] Danne C., Michaudel C., Skerniskyte J., Planchais J., Magniez A., Agus A., Michel M.L., Lamas B., Da Costa G., Spatz M. (2022). CARD9 in neutrophils protects from colitis and controls mitochondrial metabolism and cell survival. Gut.

[33] Pfaller M.A., Diekema D.J. (2007). Epidemiology of invasive candidiasis: A persistent public health problem. Clin. Microbiol. Rev..

[34] Gross O., Gewies A., Finger K., Schafer M., Sparwasser T., Peschel C., Forster I., Ruland J. (2006). Card9 controls a non-TLR signalling pathway for innate anti-fungal immunity. Nature.

[35] Trofa D., Gacser A., Nosanchuk J.D. (2008). Candida parapsilosis, an emerging fungal pathogen. Clin. Microbiol. Rev..

[36] Zuza-Alves D.L., Silva-Rocha W.P., Chaves G.M. (2017). An update on Candida tropicalis based on basic and clinical approaches. Front. Microbiol..

[37] Whibley N., Jaycox J.R., Reid D., Garg A.V., Taylor J.A., Clancy C.J., Nguyen M.H., Biswas P.S., McGeachy M.J., Brown G.D. (2015). Delinking CARD9 and IL-17: CARD9 protects against Candida tropicalis infection through a TNF-alpha-dependent, IL-17-independent mechanism. J. Immunol..

[38] Wang T., Fan C., Yao A., Xu X., Zheng G., You Y., Jiang C., Zhao X., Hou Y., Hung M.C. (2018). The adaptor protein CARD9 protects against colon cancer by restricting mycobiota-mediated expansion of myeloid-derived suppressor cells. Immunity.

[39] Voelz K., May R.C. (2010). Cryptococcal interactions with the host immune system. Eukaryot. Cell.

[40] Campuzano A., Castro-Lopez N., Martinez A.J., Olszewski M.A., Ganguly A., Leopold Wager C., Hung C.Y., Wormley F.L. (2020). CARD9 is required for classical macrophage activation and the induction of protective immunity against pulmonary cryptococcosis. mBio.

[41] Kitai Y., Sato K., Tanno D., Yuan X., Umeki A., Kasamatsu J., Kanno E., Tanno H., Hara H., Yamasaki S. (2021). Role of dectin-2 in the phagocytosis of Cryptococcus neoformans by dendritic cells. Infect. Immun..

[42] Yamamoto H., Nakamura Y., Sato K., Takahashi Y., Nomura T., Miyasaka T., Ishii K., Hara H., Yamamoto N., Kanno E. (2014). Defect of CARD9 leads to impaired accumulation of gamma interferon-producing memory phenotype T cells in lungs and increased susceptibility to pulmonary infection with Cryptococcus neoformans. Infect. Immun..

[43] Saijo S., Ikeda S., Yamabe K., Kakuta S., Ishigame H., Akitsu A., Fujikado N., Kusaka T., Kubo S., Chung S.H. (2010). Dectin-2 recognition of alpha-mannans and induction of Th17 cell differentiation is essential for host defense against Candida albicans. Immunity.

[44] Nakamura K., Kinjo T., Saijo S., Miyazato A., Adachi Y., Ohno N., Fujita J., Kaku M., Iwakura Y., Kawakami K. (2007). Dectin-1 is not required for the host defense to Cryptococcus neoformans. Microbiol. Immunol..

[45] Mansour M.K., Latz E., Levitz S.M. (2006). Cryptococcus neoformans glycoantigens are captured by multiple lectin receptors and presented by dendritic cells. J. Immunol..

[46] Mansour M.K., Schlesinger L.S., Levitz S.M. (2002). Optimal T cell responses to Cryptococcus neoformans mannoprotein are dependent on recognition of conjugated carbohydrates by mannose receptors. J. Immunol..

[47] Harris J.E., Sutton D.A., Rubin A., Wickes B., De Hoog G.S., Kovarik C. (2009). Exophiala spinifera as a cause of cutaneous phaeohyphomycosis: Case study and review of the literature. Med. Mycol..

[48] Lu X.L., Najafzadeh M.J., Dolatabadi S., Ran Y.P., Gerrits van den Ende A.H., Shen Y.N., Li C.Y., Xi L.Y., Hao F., Zhang Q.Q. (2013). Taxonomy and epidemiology of *Mucor irregularis*, agent of chronic cutaneous mucormycosis. Persoonia.

[49] Sun L., Zhang S., Wan Z., Li R., Yu J. (2021). In vivo and in vitro impairments in T helper cell and neutrophil responses against *Mucor irregularis* in Card9 knockout mice. Infect. Immun..

[50] Gigliotti F., Limper A.H., Wright T. (2014). Pneumocystis. Cold Spring Harb. Perspect. Med..

[51] Kottom T.J., Nandakumar V., Hebrink D.M., Carmona E.M., Limper A.H. (2020). A critical role for CARD9 in pneumocystis pneumonia host defence. Cell Microbiol..

[52] McGreal E.P., Rosas M., Brown G.D., Zamze S., Wong S.Y., Gordon S., Martinez-Pomares L., Taylor P.R. (2006). The carbohydrate-recognition domain of Dectin-2 is a C-type lectin with specificity for high mannose. Glycobiology.

[53] Akahori Y., Miyasaka T., Toyama M., Matsumoto I., Miyahara A., Zong T., Ishii K., Kinjo Y., Miyazaki Y., Saijo S. (2016). Dectin-2-dependent host defense in mice infected with serotype 3 Streptococcus pneumoniae. BMC Immunol..

[54] Ishizuka S., Yokoyama R., Sato K., Shiroma R., Nakahira A., Yamamoto H., Takano K., Kagesawa T., Miyasaka T., Kasamatsu J. (2020). Effect of CARD9 deficiency on neutrophil-mediated host defense against pulmonary infection with Streptococcus pneumoniae. Infect. Immun..

[55] Collins J.W., Keeney K.M., Crepin V.F., Rathinam V.A., Fitzgerald K.A., Finlay B.B., Frankel G. (2014). Citrobacter rodentium: Infection, inflammation and the microbiota. Nat. Rev. Microbiol..

[56] Lamas B., Michel M.L., Waldschmitt N., Pham H.P., Zacharioudaki V., Dupraz L., Delacre M., Natividad J.M., Costa G.D., Planchais J. (2018). Card9 mediates susceptibility to intestinal pathogens through microbiota modulation and control of bacterial virulence. Gut.

[57] Sokol H., Conway K.L., Zhang M., Choi M., Morin B., Cao Z., Villablanca E.J., Li C., Wijmenga C., Yun S.H. (2013). Card9 mediates intestinal epithelial cell restitution, T-helper 17 responses, and control of bacterial infection in mice. Gastroenterology.

[58] Lebeis S.L., Bommarius B., Parkos C.A., Sherman M.A., Kalman D. (2007). TLR signaling mediated by MyD88 is required for a protective innate immune response by neutrophils to Citrobacter rodentium. J. Immunol..

[59] Gibson D.L., Ma C., Bergstrom K.S., Huang J.T., Man C., Vallance B.A. (2008). MyD88 signalling plays a critical role in host defence by controlling pathogen burden and promoting epithelial cell homeostasis during Citrobacter rodentium-induced colitis. Cell. Microbiol..

[60] Kawasaki T., Kawai T. (2014). Toll-like receptor signaling pathways. Front. Immunol..

[61] Roth S., Ruland J. (2013). Caspase recruitment domain-containing protein 9 signaling in innate immunity and inflammation. Trends Immunol..

[62] Jo E.K. (2008). Mycobacterial interaction with innate receptors: TLRs, C-type lectins, and NLRs. Curr. Opin. Infect. Dis..

[63] Harding C.V., Boom W.H. (2010). Regulation of antigen presentation by Mycobacterium tuberculosis: A role for Toll-like receptors. Nat. Rev. Microbiol..

[64] El-Etr S.H., Cirillo J.D. (2001). Entry mechanisms of mycobacteria. Front. Biosci..

[65] Dorhoi A., Desel C., Yeremeev V., Pradl L., Brinkmann V., Mollenkopf H.J., Hanke K., Gross O., Ruland J., Kaufmann S.H.E. (2010). The adaptor molecule CARD9 is essential for tuberculosis control. J. Exp. Med..

[66] Ahmad-Mansour N., Loubet P., Pouget C., Dunyach-Remy C., Sotto A., Lavigne J.P., Molle V. (2021). Staphylococcus aureus toxins: An update on their pathogenic properties and potential treatments. Toxins.

[67] Askarian F., Wagner T., Johannessen M., Nizet V. (2018). Staphylococcus aureus modulation of innate immune responses through Toll-like (TLR), (NOD)-like (NLR) and C-type lectin (CLR) receptors. FEMS Microbiol. Rev..

[68] Herrero-Fresno A., Olsen J.E. (2018). Salmonella Typhimurium metabolism affects virulence in the host—A mini-review. Food Microbiol..

[69] Broz P., Ohlson M.B., Monack D.M. (2012). Innate immune response to Salmonella Typhimurium, a model enteric pathogen. Gut Microbes.

[70] Pereira M., Tourlomousis P., Wright J., Monie T.P., Bryant C.E. (2016). CARD9 negatively regulates NLRP3-induced IL-1beta production on Salmonella infection of macrophages. Nat. Commun..

[71] Weiser J.N., Ferreira D.M., Paton J.C. (2018). Streptococcus pneumoniae: Transmission, colonization and invasion. Nat. Rev. Microbiol..

[72] Poeck H., Bscheider M., Gross O., Finger K., Roth S., Rebsamen M., Hannesschlager N., Schlee M., Rothenfusser S., Barchet W. (2010). Recognition of RNA virus by RIG-I results in activation of CARD9 and inflammasome signaling for interleukin 1 beta production. Nat. Immunol..

[73] Roth S., Rottach A., Lotz-Havla A.S., Laux V., Muschaweckh A., Gersting S.W., Muntau A.C., Hopfner K.P., Jin L., Vanness K. (2014). Rad50-CARD9 interactions link cytosolic DNA sensing to IL-1beta production. Nat. Immunol..

[74] Mamana J., Humber G.M., Espinal E.R., Seo S., Vollmuth N., Sin J., Kim B.J. (2023). Coxsackievirus B3 infects and disrupts human induced-pluripotent stem cell derived brain-like endothelial cells. Front. Cell Infect. Microbiol..

[75] Sun C., Zhang X., Yu Y., Li Z., Xie Y. (2020). CARD9 mediates T cell inflammatory response in Coxsackievirus B3-induced acute myocarditis. Cardiovasc. Pathol..

[76] Kaya Z., Dohmen K.M., Wang Y., Schlichting J., Afanasyeva M., Leuschner F., Rose N.R. (2002). Cutting edge: A critical role for IL-10 in induction of nasal tolerance in experimental autoimmune myocarditis. J. Immunol..

[77] Myers J.M., Cooper L.T., Kem D.C., Stavrakis S., Kosanke S.D., Shevach E.M., Fairweather D., Stoner J.A., Cox C.J., Cunningham M.W. (2016). Cardiac myosin-Th17 responses promote heart failure in human myocarditis. JCI Insight.

[78] Zelaya H., Alvarez S., Kitazawa H., Villena J. (2016). Respiratory antiviral immunity and immunobiotics: Beneficial effects on inflammation-coagulation interaction during Influenza virus infection. Front. Immunol..

[79] Uematsu T., Iizasa E., Kobayashi N., Yoshida H., Hara H. (2015). Loss of CARD9-mediated innate activation attenuates severe influenza pneumonia without compromising host viral immunity. Sci. Rep..

[80] McJunkin J.E., de los Reyes E.C., Irazuzta J.E., Caceres M.J., Khan R.R., Minnich L.L., Fu K.D., Lovett G.D., Tsai T., Thompson A. (2001). La Crosse encephalitis in children. N. Engl. J. Med..

[81] Gaensbauer J.T., Lindsey N.P., Messacar K., Staples J.E., Fischer M. (2014). Neuroinvasive arboviral disease in the United States: 2003 to 2012. Pediatrics.

[82] Verbruggen P., Ruf M., Blakqori G., Overby A.K., Heidemann M., Eick D., Weber F. (2011). Interferon antagonist NSs of La Crosse virus triggers a DNA damage response-like degradation of transcribing RNA polymerase II. J. Biol. Chem..

[83] Mukherjee P., Woods T.A., Moore R.A., Peterson K.E. (2013). Activation of the innate signaling molecule MAVS by bunyavirus infection upregulates the adaptor protein SARM1, leading to neuronal death. Immunity.

[84] Hofmann H., Li X., Zhang X., Liu W., Kuhl A., Kaup F., Soldan S.S., Gonzalez-Scarano F., Weber F., He Y. (2013). Severe fever with thrombocytopenia virus glycoproteins are targeted by neutralizing antibodies and can use DC-SIGN as a receptor for pH-dependent entry into human and animal cell lines. J. Virol..

[85] Monteiro J.T., Schon K., Ebbecke T., Goethe R., Ruland J., Baumgartner W., Becker S.C., Lepenies B. (2019). The CARD9-associated C-Type lectin, mincle, recognizes La Crosse Virus (LACV) but plays a limited role in early antiviral responses against LACV. Viruses.

[86] Libbey J.E., Kirkman N.J., Smith M.C., Tanaka T., Wilcox K.S., White H.S., Fujinami R.S. (2008). Seizures following picornavirus infection. Epilepsia.

[87] Stewart K.A., Wilcox K.S., Fujinami R.S., White H.S. (2010). Development of postinfection epilepsy after Theiler’s virus infection of C57BL/6 mice. J. Neuropathol. Exp. Neurol..

[88] Pavasutthipaisit S., Stoff M., Ebbecke T., Ciurkiewicz M., Mayer-Lambertz S., Stork T., Pavelko K.D., Lepenies B., Beineke A. (2021). CARD9 deficiency increases hippocampal injury following acute neurotropic Picornavirus infection but does not affect pathogen elimination. Int. J. Mol. Sci..

[89] Castro G.R., Panilaitis B., Bora E., Kaplan D.L. (2007). Controlled release biopolymers for enhancing the immune response. Mol. Pharm..

[90] Mocanu G., Mihai D., Moscovici M., Picton L., LeCerf D. (2009). Curdlan microspheres. Synthesis, characterization and interaction with proteins (enzymes, vaccines). Int. J. Biol. Macromol..

[91] Schoenen H., Bodendorfer B., Hitchens K., Manzanero S., Werninghaus K., Nimmerjahn F., Agger E.M., Stenger S., Andersen P., Ruland J. (2010). Cutting edge: Mincle is essential for recognition and adjuvanticity of the mycobacterial cord factor and its synthetic analog trehalose-dibehenate. J. Immunol..

[92] Desel C., Werninghaus K., Ritter M., Jozefowski K., Wenzel J., Russkamp N., Schleicher U., Christensen D., Wirtz S., Kirschning C. (2013). The Mincle-activating adjuvant TDB induces MyD88-dependent Th1 and Th17 responses through IL-1R signaling. PLoS ONE.

[93] Werninghaus K., Babiak A., Gross O., Holscher C., Dietrich H., Agger E.M., Mages J., Mocsai A., Schoenen H., Finger K. (2009). Adjuvanticity of a synthetic cord factor analogue for subunit Mycobacterium tuberculosis vaccination requires FcRgamma-Syk-Card9-dependent innate immune activation. J. Exp. Med..

[94] Schweneker K., Gorka O., Schweneker M., Poeck H., Tschopp J., Peschel C., Ruland J., Gross O. (2013). The mycobacterial cord factor adjuvant analogue trehalose-6,6′-dibehenate (TDB) activates the Nlrp3 inflammasome. Immunobiology.

[95] Gantner B.N., Simmons R.M., Canavera S.J., Akira S., Underhill D.M. (2003). Collaborative induction of inflammatory responses by dectin-1 and Toll-like receptor 2. J. Exp. Med..

[96] Goodridge H.S., Simmons R.M., Underhill D.M. (2007). Dectin-1 stimulation by Candida albicans yeast or zymosan triggers NFAT activation in macrophages and dendritic cells. J. Immunol..

[97] Goodridge H.S., Shimada T., Wolf A.J., Hsu Y.M., Becker C.A., Lin X., Underhill D.M. (2009). Differential use of CARD9 by dectin-1 in macrophages and dendritic cells. J. Immunol..

[98] Xu Z., Qiao S., Qian W., Zhu Y., Yan W., Shen S., Wang T. (2022). Card9 protects fungal peritonitis through regulating Malt1-mediated activation of autophagy in macrophage. Int. Immunopharmacol..

[99] Zhang G., Meredith T.C., Kahne D. (2013). On the essentiality of lipopolysaccharide to Gram-negative bacteria. Curr. Opin. Microbiol..

[100] Nguyen D.T., de Witte L., Ludlow M., Yuksel S., Wiesmuller K.H., Geijtenbeek T.B., Osterhaus A.D., de Swart R.L. (2010). The synthetic bacterial lipopeptide Pam3CSK4 modulates respiratory syncytial virus infection independent of TLR activation. PLoS Pathog..

[101] Soares J.B., Pimentel-Nunes P., Roncon-Albuquerque R., Leite-Moreira A. (2010). The role of lipopolysaccharide/toll-like receptor 4 signaling in chronic liver diseases. Hepatol. Int..

[102] Tsolmongyn B., Koide N., Jambalganiin U., Odkhuu E., Naiki Y., Komatsu T., Yoshida T., Yokochi T. (2013). A Toll-like receptor 2 ligand, Pam3CSK4, augments interferon-gamma-induced nitric oxide production via a physical association between MyD88 and interferon-gamma receptor in vascular endothelial cells. Immunology.

[103] Grimes C.L., Ariyananda Lde Z., Melnyk J.E., O’Shea E.K. (2012). The innate immune protein Nod2 binds directly to MDP, a bacterial cell wall fragment. J. Am. Chem. Soc..

[104] Chassaing B., Aitken J.D., Malleshappa M., Vijay-Kumar M. (2014). Dextran sulfate sodium (DSS)-induced colitis in mice. Curr. Protoc. Immunol..

[105] Lamas B., Richard M.L., Leducq V., Pham H.P., Michel M.L., Da Costa G., Bridonneau C., Jegou S., Hoffmann T.W., Natividad J.M. (2016). CARD9 impacts colitis by altering gut microbiota metabolism of tryptophan into aryl hydrocarbon receptor ligands. Nat. Med..

[106] Malik A., Sharma D., Malireddi R.K.S., Guy C.S., Chang T.C., Olsen S.R., Neale G., Vogel P., Kanneganti T.D. (2018). SYK-CARD9 signaling axis promotes gut fungi-mediated inflammasome activation to restrict colitis and colon cancer. Immunity.

[107] Li Y., Liang P., Jiang B., Tang Y., Lv Q., Hao H., Liu Z., Xiao X. (2019). CARD9 inhibits mitochondria-dependent apoptosis of cardiomyocytes under oxidative stress via interacting with Apaf-1. Free Radic. Biol. Med..

[108] Di Marzo N., Chisci E., Giovannoni R. (2018). The role of hydrogen peroxide in redox-dependent signaling: Homeostatic and pathological responses in mammalian cells. Cells.

[109] Kim K.H., Kabir E., Kabir S. (2015). A review on the human health impact of airborne particulate matter. Environ. Int..

[110] Zhu Q., Liu X., Wu H., Yang C., Wang M., Chen F., Cui Y., Hao H., Hill M.A., Liu Z. (2023). CARD9 deficiency improves the recovery of limb ischemia in mice with ambient fine particulate matter exposure. Front. Cardiovasc. Med..

[111] Zhao Q., Chen H., Yang T., Rui W., Liu F., Zhang F., Zhao Y., Ding W. (2016). Direct effects of airborne PM2.5 exposure on macrophage polarizations. Biochim. Biophys. Acta.

[112] Jia X.M., Tang B., Zhu L.L., Liu Y.H., Zhao X.Q., Gorjestani S., Hsu Y.M., Yang L., Guan J.H., Xu G.T. (2014). CARD9 mediates Dectin-1-induced ERK activation by linking Ras-GRF1 to H-Ras for antifungal immunity. J. Exp. Med..

